# Family adjustment and resilience after a parental cancer diagnosis

**DOI:** 10.1007/s00520-024-08608-x

**Published:** 2024-06-04

**Authors:** Katarina Perak, Fiona E. J. McDonald, Janet Conti, Yi Sing Yao, Xiomara Skrabal Ross

**Affiliations:** 1 School of Psychology, Western Sydney University, Sydney, Australia; 2 Research, Policy & Patient Department, Canteen Australia, Sydney, Australia; 3 Faculty of Medicine and Health, The University of Sydney, Camperdown, Australia; 4 Translational Health Research Institute, Western Sydney University, Sydney, Australia; 5 Cancer and Palliative Care Outcomes Centre, Queensland University of Technology, Brisbane, Australia

**Keywords:** Family resilience, Parental cancer, Psychosocial, Family system, Parent experience

## Abstract

**Purpose:**

To explore the resources, parents with cancer and their partners draw upon to sustain their family resilience through the cancer experience.

**Methods:**

Fifteen participants who were parents of children aged 8 to 25 years completed phone, audio-recorded, and semi-structured interviews. Of these participants, 11 were parents diagnosed with cancer, and four were partners of a parent diagnosed with cancer. Interview questions aimed to increase understanding about how families communicate, connect, and face challenges from the cancer experience. Interview data was analysed using inductive thematic analysis to provide scope to generate themes from parent’s experiences rather than to test pre-existing frameworks.

**Results:**

The thematic analysis of interview transcripts generated three key themes related to family resilience: (1) adaptability to changes in roles and routines, (2) open communication within the family, and (3) accepting support from others.

**Conclusion:**

This study found that parents’ ability to use personal resources when faced with significant challenges helped to improve the resilience of parents’ family system. Further research is needed to understand the factors that influence family resilience when a parent is diagnosed with cancer. Implications for the development of targeted interventions that provide support to not only the patient, but their whole family system will be discussed.

**Supplementary Information:**

The online version contains supplementary material available at 10.1007/s00520-024-08608-x.

## Introduction

The global prevalence of cancer signifies a substantial health challenge. In 2020, worldwide, there were approximately 19 million new cancer cases and nearly 10 million cancer-related deaths [1]. As being diagnosed with cancer not only affects the individual but also extensively impacts the entire family system, it is often termed a ‘family disease’ [2, 3]. In Australia, nearly one in two adults will be diagnosed cancer in their lifetime, with approximately 18% diagnosed whilst raising children under 18 years [4, 5]. Additionally, nearly 10,000 parents are diagnosed with cancer every year, affecting approximately 21,000 Australian adolescents and young adults (AYA) aged 12–25 annually [6, 7].

Current evidence primarily focuses on family resilience in paediatric cancer settings, concentrating on the importance of the parental role in family resilience [8, 9]. Some of this literature also explores the psychosocial impact on parents and their resilience post-paediatric cancer [10, 11]. Limited studies however examine family resilience dynamics in households where a parent faces cancer [12]. Whilst parents diagnosed with cancer experience substantive stressors [13], literature has largely focused on the impact of parental cancer on children, neglecting the challenges parents and/or their partners encounter whilst managing post-diagnosis stressors alongside their parental duties [14, 15]. For instance, a study on family resilience explored how parental cancer affects children, revealing that communication between AYA and parents, as well as AYA’s perceived stress significantly influenced family resilience especially among late adolescents [12]. The emphasis on the impact of parental cancer on children rather than the challenges faced by parents and/or their partners highlights a significant gap in understanding family resilience especially when a parent faces cancer.

Families struggling with a cancer diagnosis often experience heightened distress and compromised cohesion [16]. Family resilience plays a crucial role in mitigating these challenges [16]. Defined as “maintaining stable and healthy functioning following distressing events” [17], family resilience differs from individual resilience in that it pertains to a family unit’s ability to adjust, persevere, and potentially grow through adversity [18]. A systemic approach to family resilience underscores the influence of the family’s strengths and resources in coping with, and navigating, significant life stressors like cancer [19, 20]. Enhancing family resilience not only mitigates the impacts of crises like cancer but also improves the psychological wellbeing, disease trajectory, and outcomes for the affected individuals [21–23]. Studies stress the parental role as crucial in fostering family resilience [24]. A cancer diagnosis can spark a reconceptualization of parental values causing significant shifts in the family structure [25]. A parent’s ability to be adaptive to these changes, as well as utilising personal resources, significantly enhances family resilience amid adversity [26].

In the context of the significant number of families affected by cancer [4], this study aims to identify the resources that parents with cancer and their partners draw upon to sustain their family resilience during a parental cancer experience. Understanding these dynamics has scope to contribute to the existing knowledge gap on how families facing parental cancer navigate and respond to adversity and inform future research and interventions to support families affected by parental cancer.

## Method

### Study design

The study has a cross sectional qualitative design with semi-structured interviews to explore how parents and their partners communicated, connected, and faced challenges from the cancer experience. Participants engaged in phone, audio-recorded semi-structured interviews, with average duration of 40 min. Interview scripts were structured based on Walsh’s family resilience framework [17, 18, 27–29], which cites family efficacy, optimism for the future, flexibility, and social capital as predictors of resilience. The framework further suggests that communication and problem-solving are additional predictors of family resilience [17, 18, 27–29].

Participants were asked about various aspects of their family’s cancer experience, including their family communication, connectedness, making meaning of adversity, and maintaining a positive outlook. These questions aimed to understand how families coped with cancer, foster resilience, and reveal coping strategies. The interview guide is provided in Supplementary File 1.

### Participants

A total of 15 parents who had been either diagnosed (11 parents), or their partner diagnosed (4 parents) with cancer and had children aged 8 to 25 years participated in this study. Evidence suggests that parents with cancer who have dependent children experience considerably higher distress and face worse psychological outcomes, compared to patients without dependent children [30–32]. As such, we included parents with children up to 25 years of age, acknowledging that young adults within this range might still be studying or reliant on their parents for support. This study was part of a longitudinal study with a sample of parents of children 8–25 years old. Participants in the longitudinal study (parent with cancer diagnosis, partner, and children) completed surveys that we deemed children 8 years and older would be able to understand without assistance. Ethics approval was gained from the Western Sydney University (WSU) Human Research Ethics Committee approval (RH14962) and the Hunter New England Human Research Ethics Committee (17/07/19/4.03).

### Materials and procedure

The interviews were conducted by XSR between March 2021 and March 2022. Interviews were transcribed verbatim and de-identified, including replacing names with participant numbers. Interviews were coded using NVivo software [33].

### Data analysis

Data was analysed with inductive, reflexive thematic analysis [34, 35]. This approach aimed to elucidate participants’ experiences, representations, views, and perceptions regarding a given phenomenon [36]. Analysis involved an inductive approach rather than testing a pre-existing framework, allowing the data content to guide coding and theme development, which facilitated a nuanced and implicit interpretation, crucial when exploring under-researched phenomena [34]. Methodological rigour was maintained by meticulously documenting each stage of the coding and theme development process.

Initial readings of interview transcripts were followed by the generation of diverse codes (by KP in discussion with JC) capturing crucial data facets such as “emotional connection”, “close relationship with children”, and “gratitude”. These codes were iteratively revised, consolidated, and refined in consultation with FM and XSR to ensure agreement and discuss theme development. This practice is beneficial in fostering opportunities for rich data analysis [37]. Whilst each participant had a unique cancer experience, overarching commonalities emerged. Subthemes were employed to encapsulate similarities and differences across shared central concepts. The final analysis phase involved a comprehensive exploration of themes and subthemes aligning with the study’s aim to explore the experiences and resources that sustain families when confronted with parental cancer diagnoses. This included an in-depth examination, selection, and analysis of exemplar quotes to illuminate each theme and subtheme.

## Results

Of the 15 participants, the majority were female (13/15). Most had two to three children (14/15). At the time of the interview, most participants had completed their treatment (10/15), and patients reported a variety of cancer diagnoses. Table 1 outlines the primary sociodemographic and clinical characteristics of these participants.

**Table 1:** Summary of sociodemographic and clinical characteristics of participants

Participant ID	Cancer patient (CP) or partner (P)	Gender (M) or (F)	Type of cancer	Current treatment stage	Number of children	Country of birth
P1	Cancer patient	Male	Bronchus and lung	Finished treatment	2	Australia
P2	Partner	Female	Rectum	Finished treatment	2	Australia
P3	Cancer patient	Male	Rectum	Finished treatment	3	Australia
P4	Cancer patient	Female	Breast	Finished treatment	3	Australia
P5	Cancer patient	Female	Bowel	Finished treatment	3	Other
P6	Cancer patient	Female	Breast	Finished treatment	3	Australia
P7	Partner	Female	N/A	Finished treatment	2	Australia
P8	Cancer patient	Female	Breast	Surveillance and maintenance treatment	3	Australia
P9	Cancer patient	Female	Breast	Undergoing treatment	2	Australia
P10	Partner	Female	Melanoma	Undergoing treatment	2	Australia
P11	Cancer patient	Female	Melanoma	Surveillance and maintenance Treatment	2	Australia
P12	Partner	Female	Bowel	Deceased	3	Australia
P13	Cancer patient	Female	Breast	Finished treatment	2	Australia
P14	Cancer patient	Female	Breast	Finished treatment	1	Australia
P15	Cancer patient	Female	Rectum	Finished treatment	3	Other

*CP*, cancer patient; *P*, partner; *M*, male; *F*, female

### Thematic analysis

**Fig. 1:**
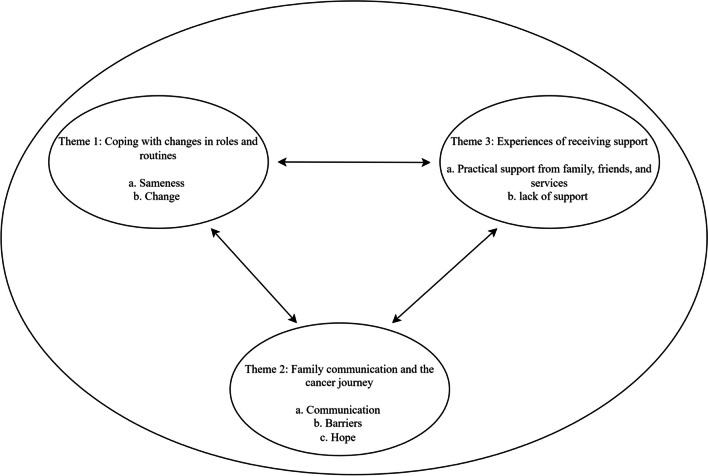
Thematic map of adjustment and resilience to living with parental cancer

The thematic analysis of parent experiences generated three primary themes, that related to family adjustment and resilience in the face of parental cancer experiences: (i) coping with change in roles and routines; (ii) family communication about the cancer journey; and (iii) experiences of receiving support. The inter-relationship between themes is depicted in the thematic map (Fig. 1), and key participation quotations for each of themes are described under each theme. Additional quotations are summarised in Supplementary File 2.

- Theme 1: Coping with change in roles and routines

All participants talked about how their or their partners’ parental cancer diagnosis impacted their family system, with most of them (13/15) describing changes to family routines and roles. This included adapting to different roles (caregiver/provider) and adjusting and re-prioritising routines around illness and treatment. Two parents also spoke about the steps that they took to keep things “normal” in their family roles and routines, seeking to maintain the same roles and routines that they had prior to the cancer diagnosis.

- Change

Five mothers diagnosed with cancer and in heterosexual relationships expressed that the adverse physical effects of cancer and its treatment led their partners to take on more of a traditional motherhood/caregiver role within the family system. These women described how their partners demonstrated flexibly and tenacity in taking on new roles whilst still maintaining their traditional role as providers. The following participant witnessed a closeness between her partner and their children during this time of change.

“My husband’s become much closer to the kids. He saw his role as more of the breadwinner. He would go out and earn the money, and that was his contribution to the family. Whereas he takes a lot more time to do family stuff now” P4 (CP, F)

Likewise, when fathers were diagnosed with cancer, their partners took on both the caregiver and provider role, shifting roles to reallocate power in the family that had previously been assumed to be the domain of their partner. Parents tended to speak about these role shifts in a matter-of-fact way that is, this role was what was required to sustain their family.

“I went from being the nurturer/gatherer sort of role to the whole alpha role” P12 (P, F)

This suggests that flexibility for the partner to take on both the caretaker and provider roles is key in maintaining a family’s ability to function, adapt, and respond to adversity.

Children also shifted into different roles within the family. Six parents noted their children became more “independent”, with some becoming more involved with household chores (washing clothes and dishes) and some older children finding employment.

Interestingly, there appeared to be an unspoken and natural shifting of roles within the family system. Children were observed to attune to needs of their ill parent, demonstrating an ability to adjust their expectations, take on more responsibility, and become a support system for their parents. These parents highlighted an ability to be vulnerable, to acknowledge limits (due to illness), and to be courageous enough to lean on others. The collective impact of a parental cancer was described by one father “I think it’s important to acknowledge that this is a shared difficulty” (P1) and thus, all members must shift together when responding to adversity. This was exemplified by P2:

“They took on more independence, they took on more roles that enabled them to do more around the house, they weren’t quite so dependent. They learned how to chuck on a load of washing, they learned how to turn the dishwater on, not just unpack it. One of them went out and got himself a job, and then, later on, encouraged his brother to go and get a job” P2 (P, F)

b) Sameness

Two parents expressed a commitment to maintaining their traditional roles that they had established prior to the parental cancer diagnosis. Implicit in this “business as usual” approach that these parents took during their cancer journey was an effort to hold onto stability and normality during a time of uncertainty.

“So, nothing changed there. I would take the kids to school and then get on the transport bus that took us from - because we were in (Town 1) and the radiation treatment was in (Town 2). Like I say, not a lot changed as far as things being business as usual” P3 (CP, M)

- Theme 2: Family communication about the cancer journey

With the systemic effects of cancer on families, some parents appeared to value open communication throughout the cancer journey. Others expressed difficulties with communication within the family system.

- a) Communication

For most of the parents interviewed (12/15), having an open communication style allowed the family to cope with a parental cancer diagnosis. Open communication created a safe space for family members to navigate the emotions related to cancer and the vulnerability of parents’ emotional exposure fostered deeper family connections. Openly discussing the cancer with children served as a vital resource for them to understand and respond to the family’s needs as it is challenging for children to adapt to a situation they are unaware of.

“We’re very open in our communication about it, and my husband and I actively chose to model that ourselves consciously expressing how we feel even if it’s that we’re sad or overwhelmed or whatever, so to create a space where the kids can share their emotions openly” P15 (CP, F).

- b) Barriers

Some parents noted that family members struggled to communicate their distress in response to the cancer experience. This reluctance to discuss the cancer caused distress within the family. Partners of fathers with cancer actively avoided sensitive topics related to the illness. This pattern may reflect gendered communication tendencies, with women more inclined to talk about the cancer than men. One woman identified these communication challenges as a source of additional stress alongside dealing with the cancer. Whilst the intention was to minimise distress, avoiding difficult conversations ultimately heightened anxiety and isolation within the family.

“Probably we have more transaction communications. So, I suppose with [name of partner], there’s lots of off-limits subjects that I don’t go near, because they make him angry or sad. [Name of partner] and I had, and continue to have, marital/relationship issue, as a result of that” P7 (P, F).

c) Hope

A total of 13 parents discussed how their relationship with hope also sustained their families through the challenges faced along their cancer journey. Parents discussed the dynamic relationship they had with hope, and how hope appeared in different forms such as hope for recovery, hope for new treatment options, and hope for just a little bit more time with their family. For these parents, hope provided an opportunity to let go of the uncertainty of a cancer diagnosis. By accepting the lack of control, they had over their cancer journey, parents were able to anchor to the “now”.

“You get a choice, in my view, whether you look at the good side or the bad side. […] I think that if you’ve got hope you – a whole lot of other things fit together. Once you lose hope you stop doing a whole range of things and then you start falling down into a darker place” P10 (P, F).

On the other hand, six parents diagnosed with cancer described how others’ positive attitudes about their cancer prognosis caused significant frustration. Uncertainty is an inherent aspect of a cancer diagnosis and can bring about fears around treatment, pain, and survival. These fears are amplified for a parent diagnosed with cancer, as their difficult journey is a shared burden for their family. These parents felt their worries were invalidated when others had an “overly positive” approach in relation to their cancer. For these parents, an overly positive approach did not help with uncertainty. A mother (P12) explained that “whilst hope is a fantastic thing, hope is not a promise”.

“When I’m feeling like rubbish, and—sometimes I just felt like the positivity—it was like they were putting on a show, because I wasn’t feeling that inside. But they were all so upbeat that sometimes I was like, you can acknowledge that it’s not all positive and high hopes. It is horrible, and I may not get through it. But just to have that acknowledged would have been nice” P4 (CP, F).

- Theme 3: Experiences of receiving support

Each parent in this study talked about the importance of receiving support from others to sustain family functioning. Whilst some parents received support from family members, friends, and services, other parents spoke about their disappointment with the type or lack of support that they received.

- a) Practical support from family, friends, and services

The parents highlighted the importance of receiving support from family, friends, and services during their cancer journey. Grandparents were a key support system which acted as a relief for the unwell parents and provided stability for the rest of the family. One mother (P15) with cancer reported that her parents kept her children’s “lives going”. This support did not buffer all family challenges but provided the family system enough resources to overcome everyday life changes.

Five parents spoke about the important role that schoolteachers played in supporting their family in their cancer journey, acting as a safeguard for their children.

Engagement with mental-health support and services was a common experience for parents with cancer. This included interactions with counsellors, psychologists, GPs (providing anti-depressants), chaplains, and social workers. Seeking support from people independent of their family provided family members with space to process emotions outside of the family system.

“Having mum and dad here to pick up the pieces where we left off, with school drop-off and making lunches, things like that when I wasn’t quite well enough to do the normal things. It was just really important. We really would have struggled without that kind of really close family support… I saw a psychologist at my radiation […] I could speak to him about all of these things, and he was just, I suppose somebody who wasn’t involved in it all, could just be that person that I could vent to, without putting stress on anyone or worrying anybody” P4 (CP, F).

“Their support was amazing. The teachers would ring me every week to check in. They kept an eye on the kids. They offered – yeah, they talked to the children” P6 (CP, F).

- b) Lack of support

Some parents were disappointed in the lack of support they received from family, friends, and services. Feelings of pain and isolation resonated in these parents’ accounts when they spoke about the little to no support they received from their community. These parents trusted their community to support their family through a crisis however support did not eventuate.

“Initially, people offered, “Oh, let me know if I can help out”, “I’ll come and clean your house,” that sort of thing. But when it actually came—push came to shove, nothing actually eventuated. The lack of support from our church family was actually quite astounding and actually quite—It actually made us all feel a bit hurt” P2 (P, F).

## Discussion

Despite the complex challenges faced by parents with a cancer diagnosis and their partners [38], common resources sustain their families through this journey [26]. This study highlighted three key resources supporting the resilience of families facing parental cancer diagnoses. Firstly, family functioning relies on flexibility in parental roles and routines during a cancer diagnosis. Stability in fulfilling family roles, regardless of caregiving or provider roles, is crucial for adaptation and response to adversity. This aligns with existing literature that underscores the significance of role flexibility in maintaining stability and in reducing family distress [18, 39]. Our study also touched on the dynamic nature of communication and vulnerability between ill parents and their children. Acknowledging caregiving limitations due to illness fostered independence and caring attitudes in children, unlike families where pre-diagnosis roles were rigidly maintained. Furthermore, vulnerable conversations centred around hopes for future may have served as a tool to unite family members in the face of adversity.

Related to this, secondly, the study findings suggest that open communication within families strengthened feelings of connectedness and emotional safety within the vulnerability that a cancer diagnosis poses for a family system. This aligns with existing literature, in that when faced with a parental cancer diagnosis, open communication amongst family members had been found to lower psychological distress and increase coping and adaptability to illness-related difficulties [40]. Our study also found that fathers living with cancer often faced challenges in effectively communicating about their illness, that was perceived to contribute to higher levels of family distress and isolation. This is consistent with previous research that showed a tendency among men with cancer to avoid discussing their diagnosis to not cause distress in their partners [41].

Third, this research underscored the importance of receiving support from others to sustain family functioning. A cancer diagnosis disrupts a parent’s ability to manage their own well-being and attend to their family’s needs [42]. In our research, willingness to rely on external support helped parents manage their responsibilities, provided stability in daily activities, and allowed the parent living with cancer to focus on healing. This aligns with previous studies showing that accepting support from others help parents to adjust to adversity and cope with challenges [32, 43].

### Limitations of the study

To our knowledge, this is the first study to explore how families with a parental cancer diagnosis function, adapt, and respond to adversity. It represents a step in understanding the risk and protective factors involved in family resilience within this context of a parent living with cancer.

The study acknowledges limitations, such as a gender disparity in participants (mostly mothers, 13/15) and potential biases in single interviews which limit exploring change over time These interviews may be susceptible to biases such as reporting “safe” answers, potentially underreporting distress, and oversimplifying complexity [44]. Additionally, conducting interviews several months post-cancer diagnosis (at least 9), with many having completed treatment might impact participants’ recall abilities. Interpretations should be contextualised within the retrospective nature of storytelling post-cancer treatment.

Finally, this study was exploratory, and the themes that have been generated require further research and testing with a wider group of parents who are living with cancer and their partners, including males and those of diverse cultural backgrounds, living in a range of family contexts, and being treated with different health systems than Australian.

### Future directions and clinical implications

Given the magnitude of families affected by cancer, it is critical for research to focus on identifying the risk and protective factors for family resilience to help families heal and strengthen. This study highlights the need for interventions to be multi-targeted in supporting parents to communicate and build resources both within and outside the family. This will inform more targeted interventions and the provision of support to not only the patient, but their family system [16].

Future research is needed to explore the importance of vulnerable communication within a family system in the context of a parental cancer diagnosis. For example, there is a need for further research to better understand whether vulnerable communication may act as a moderator in resilience and adjustment in their own or their partner’s cancer diagnosis and treatment. Furthermore, expanding the participant group to include a more diverse group of parents, and qualitative studies with a longitudinal design has scope to inform ways to address the diverse challenges faced by parents living with cancer or a partner with cancer.

## Conclusion

This study revealed that family resilience is enhanced when parents effectively leverage internal and external resources in confronting significant challenges. Three key factors contributing to family resilience include adaptability to role and routine changes, open family communication, and the acceptance of support from others. Despite acknowledged limitations, the study accomplished its primary objective of investigating the experiences and resources that sustain families facing a parental cancer diagnosis. The need for further research focusing on parents experiencing cancer across various stages of their journey is emphasised. This includes exploring moderators and mediators of family resilience to enable targeted and comprehensive interventions that meet the needs of parents living with cancer and their partners to promote family resilience amid adversity.

### Supplementary Information

Below is the link to the electronic supplementary material.

Supplementary file1 (DOCX 33 KB)

Supplementary file2 (DOCX 32 KB)

## Data Availability

The datasets generated and analysed during the current study are not publicly available due to containing information that could compromise the privacy of research participants but are available from the corresponding author on reasonable request.
