# Supplementary material for: Family adjustment and resilience after a parental cancer diagnosis
Source: Support Care Cancer. 2024 Jun 4;32(7):409. doi: 10.1007/s00520-024-08608-x (PMC11150317; doi:10.1007/s00520-024-08608-x)
Supplement: Supplementary file 1 — Supplementary file1 (DOCX 33 KB) [file 520_2024_8608_MOESM1_ESM.docx]

**Family Resilience Study After a Parental Cancer Diagnosis**

**Time 3 Interview Script**

Thank you for your interest in participating in the interview.

The purpose of this interview is to increase understanding about how your family communicated, connected and faced challenges from the cancer experience. I’ll ask some questions about these topics.

The interview will be audio-recorded so I can listen to you and not try to make notes at the same time. The duration of the interview will be approximately 40 minutes.

After our interview I’ll upload the recording into Canteens electronic database (user and password protected) and I’ll delete it from the audio-recorder. The interview will be typed out by an external company and your name will be removed and replaced with a code and people outside of the research team will not be able to match your answers to your name.

We will then review your responses along with other people’s responses and see if we can identify common themes that come up.

If you feel discomfort during or after the interview please let me know. We can stop the interview if needed and I can refer you to an appropriate person or service^[[1]](#footnote-2)^ if you need to talk to someone about how you feel.

Do you have any questions about the interview or the study? (address questions)

**Introduction:**

Can you tell me a bit more about the cancer situation in your family? (who was diagnosed with cancer, when, is treatment active?).

Please tell me about any challenges you or your family faced (of any type) in the past?

How did you deal with it?

**Interview**

*Communication*

For most families included in our study, communication was important during difficult time.

Was communication important in your family?

Why?

Was communication in your family different over time?

Were there any challenges with communication in your family? (could you please tell me more about these challenges?)

How is communication different between different family members?

*Connectedness*

How would you describe the relationships within your family? (e.g. friendly, warm)

Do you think the relationships within your family changed after the cancer diagnosis?

What support were you able to give to others within your family?

What support did you receive?

Who supports you in your family?

Who supports your [mom, dad, sibling, partner, children]?

Do you feel like people in your family hear you when you are expressing concerns?

Who supported you outside of your family? (e.g. friends, school, neighbours)

How supported did you feel by people outside of your family? (e.g. friends, school, neighbours)

Do you generally get support from your family or outside your family?

Were you able to give people outside of your family (e.g. friends, school, neighbours) any support during this time?

*Making meaning of adversity*

How did your family react after the cancer diagnosis?

What difficulties did your family experience as a result of cancer? How did your family cope with these challenges?

*Maintenance of a positive outlook*

How did your family view the cancer experience (e.g. diagnosis, treatment)? (were they expressing hope/optimism?)

Was hope/being positive important for you? Why?

How do you think your family will react if another problem comes up?

Concept definitions:

*Connectedness*

Connectedness is the difficult balance of unity, mutual support, and collaboration within the family unit while still respecting the separateness and autonomy of the individual (Walsh, 1998). Mutual support, collaboration, and commitments are necessary for individuals and their families to survive adversity, while still respecting individual needs, differences, and boundaries.

FRAS questions: Our friends value us and who we are - We feel taken for granted by family members - We keep our feelings to ourselves - We seldom listen to family members concerns or problems - We show love and affection for family members - We think we should not get too involved with people in this community

*Making meaning of adversity*

Making meaning of adversity: Whenever an adversity or crisis situation occurs within a family system, the bedrock upon which that family unit is built is rocked. The event is something that is outside of a family’s normal realm of experiences (Hoff, 2001). Finding a way to make sense of the experience as a family system can influence a family’s reconstruction and healing. This is crucial for resilience. When making meaning of adversity, these families are also able to “normalize and contextualize distress,” by enlarging their perspectives and are able to “see their reactions and difficulties as understandable in light of a painful loss or daunting obstacle” (Walsh, 1998).

FRAS questions: The things we do for each other make us feel part of the family- We accept stressful events as part of life - We accept that problems occur unexpectedly

*Maintenance of a positive outlook*

Maintenance of a positive outlook: Resilient families must have a sense of hope for the future, regardless of how bleak their current situation may be. During the process of resilience, families are able to hold an optimistic view as opposed to a pessimistic view that they will overcome the adverse situation that they are currently in. Without this hopeful, optimistic viewpoint, families will feel hopeless that their situation cannot improve, robbing them of meaning and purpose. However, families must be reinforced by successful experiences and a nurturing community context to sustain a hopeful, optimistic viewpoint (Walsh, 1998). This reinforcement may come from one’s personal courage or through encouragement given by others. When others witness one’s courage, their own sense of courage may be inspired, facilitating a previously absent optimistic and hopeful view.

FRAS questions: We believe we can handle our problems - We can solve major problems - We can survive if another problem comes up - We feel we are strong in facing big problems - We have the strength to solve our problems - We trust things will work out even in difficult times

1. Lifeline: 131114, Cancer Council Helpline: 13 11 20, Parentline: 1300 1300 52, Canteen Online (young people) [↑](#footnote-ref-2)
