# Supplementary material for: Family adjustment and resilience after a parental cancer diagnosis
Source: Support Care Cancer. 2024 Jun 4;32(7):409. doi: 10.1007/s00520-024-08608-x (PMC11150317; doi:10.1007/s00520-024-08608-x)
Supplement: Supplementary file 2 — Supplementary file2 (DOCX 32 KB) [file 520_2024_8608_MOESM2_ESM.docx]

**Supplementary File 1. Additional** **Key Participation Quotations from Major Coded Themes**

| Coded Subthemes | Participant Quotations |
| --- | --- |
| **Theme 1 - Coping with Change in Roles and Routines** | |
| - Change |  |
| Mothers |  |
|  | “I think my husband gained more knowledge and exposure to what’s required as far as looking after three children and how to approach things with the children as well. […] Mainly the fatigue is probably the biggest impact that I've had longer term. So, I guess the challenge there was around them needing to take over the household responsibilities and looking after the children, those sorts of things” P8 (CP, F) |
|  | “My husband did every – you know, he took on a lot more of the – he took on everything. He took on the parenting during the really –stuff when I was recovering from surgery, going through treatment he did all of the household stuff, he did all the running around with the girls” P13 (CP, F) |
| Fathers | “From my wife a very substantial amount of practical support, there have been points of being very fatigued, post-surgery, or post treatment, that have meant that I’m trashed and so she’s done the heavy lifting with the family” P1 (CP, M) |
| Children | “They were a little less demanding of my time and attention for a little while there. Even though I was there, they were happy just to lay on the couch and watch TV with me” P4 (CP, F) |
|  | “My youngest son wanted to care for me and fix me, so bring me water and bring me soft toys and massage my back. Oh, just heartbreaking. I would lay in bed sometimes when I was really, really sick and he would just come in and out, in and out, bringing me things to make me better” P11 (CP, F) |
|  | “My eldest daughter, she stepped up into the role of mum - I took over the total running of the household and became the provider/protector and yeah my daughter stepped up to fill the gap that I’d left” P12 (P, F) |
| **Table 2 continued** |  |
| **Coded Subthemes** | **Participant Quotations** |
| - Sameness |  |
| Keeping family roles same prior to cancer diagnosis | “I tried to keep things as normal as possible. So, even if I wasn't really feeling up to it, I would still get up and make lunches. There was no real upheaval to anyone's life. Everything sort of just went along as it usually did” P4 (CP, F) |
| **Theme 2 - Family Communication About the Cancer Journey** | |
| - Communication | “I think that’s probably one of the most useful bits, that we have quite an open relationship in terms of talking with each other and sharing things. You’d prefer you didn’t have to talk about that with your kids, but that’s one of the silver linings. That idea that it’s okay and, in fact, important to talk about this stuff, and then seeing adults as vulnerable frail humans is not a bad thing” P1 (CP, M) |
|  | “Communication has never been a problem. It was to give them time to process what they needed to process, in the timeframes that they had within themselves to process it” P3 (CP, M) |
| - Barriers | “The youngest daughter did “close down” once the cancer arrived. You might not see it if you didn’t know her before – she was always so open and bubbly. But since the cancer she is less likely to disclose information” P5 (CP, F) |
|  | “Towards the end I found that I didn’t want to upset [name of partner] too much. […] We’d try to avoid key words that we knew would upset or anger him. We would have picked and guarded conversations at times so as not to inflame the situation. With the kids I suppose they probably copped the end result of that as well, with having a lot more angst and anxiety in the house” P12 (P, F) |
| - Hope | “I found too much positivity difficult. Where people would just say, “Well, it’s early breast cancer. You'll be fine.” And what they didn't necessarily realise was that the breast cancer that I had didn't fit the norm and had much higher risk of things not going well and all of those sorts of things. So, being positivity where, without a basis, I didn't like” P8 (CP, F) |
| **Table 2 Continued** |  |
| **Coded Subthemes** | **Participant Quotations** |
|  | “At the time I found it frustrating when I expressed fears and things and they were overly positive. […] I felt that I wasn’t always being heard because they were overly hopeful and positive” P11 (CP, F) |
|  | “I – yeah, struggle with the concept of toxic positivity, and don’t really believe that if you think everything’s going to be great it will be great” P15 (CP, F) |
| How relationship to hope sustained families through the challenges | “Well, why is it important? Because it enables you to engage with the positive things in your life, and continue to have good relationships with people, and be able to enjoy whatever time you’ve got left; the quality of life, to me, is very important” P7 (P, F) |
|  | “I think staying hopeful, focusing on the small things, being grateful for any small thing you can find, really helps you to stay grounded and to stay in the present. And stay in the present I think is really important for not allowing your emotions and everything to get out of control and get to a place where you can’t parent well and you can’t see clearly enough to sort of have perspective and be calm and be present for the children” P13 (CP, F) |
| **Theme 3 - Experiences of Receiving Support** | |
| - Practical Support from Family, Friends and Services |  |
| Grandparents | “My parents also jumped in and got quite involved and made sure that they were around a lot more for the children” P8 (CP, F) |
|  | “I think my dad – I think he sort of– you would call it like cotton wool, surrounding me kind of a thing. Where he’s, “Don’t do that. I can do that for you,” or come over and fix that and so he’s very much all the time, “Don’t lift heavy things.” P14 (CP, F) |
| **Table 2 Continued** |  |
| **Coded Subthemes** | **Participant Quotations** |
| School teachers | “So, I would - the school is very good. I mean we consider them close friends now, a lot of the teachers, and his aid - we'd call her his school mum. I'm his mum at home, and then he's got his mum at school” P4 (CP, F) |
|  | “The teacher, class teachers were certainly looking out for both girls and communicating with me if there was any issues” P13(CP, F) |
| Mental Health Support | “I think part of it was trying to make sure there was independent people to speak to […] she has engaged with a therapist about both her mental health struggles, and the trials and tribulations of being a teenager, and my cancer” P1 (CP, M) |
|  | “Youngest daughter mainly got support from outside the immediate family. Canteen was extremely helpful for her – counselling plus two camps.” P5 (CP, F) |
| - Lack of Support | “I'm sitting here trying to think of the best way of saying there wasn't a lot of support and being gracious and polite about it. I expected more support from the parish” P3 (CP, M) |
|  | “So, some people would offer support as a sort of blanket offer of “Tell me how I can help” in which case most of the time you didn't actually end up receiving any support” P8 (CP, F) |
|  |  |
